# The effect of amine-free initiator system and polymerization type on long-term color stability of resin cements: an in-vitro study

**DOI:** 10.1186/s12903-022-02456-z

**Published:** 2022-09-24

**Authors:** İdris Kavut, Mehmet Uğur

**Affiliations:** Department of Prosthodontics, Faculty of Dentistry, Yuzuncu Yil University, Van, Turkey

**Keywords:** Color stability, Amine-free, Polymerization type, Resin cement

## Abstract

**Background:**

This in vitro study evaluated the effect of amine-free initiator system and polymerization type on long-term color change of amine-free light-cure and dual-cure resin cements.

**Methods:**

Sixty disk-shaped specimens (10 × 1 mm) were prepared from six different amine-free resin cements; NX3 Nexus light-cure (LC) and dual-cure (DC), Variolink Veneer (LC) and Variolink II (DC), Relyx Veneer (LC) and Rely X Ultimate (DC). A feldspathic porcelain specimen (12 × 14 × 0.8 mm) was obtained from a CAD/CAM block (Cerec Blocks; Sirona Dental Systems GmbH, Bensheim, Germany) for color testing. The feldspathic specimen was placed on the resin cement disk and all measurements were performed without cementation. A spectrophotometer was used for color measurements. Specimens were subjected to thermal aging (5 °C and 55 °C; 5000 and 20,000 cycles). Specific color coordinate differences (ΔL, Δa, and Δb) and the total color differences (ΔE_00_) were calculated after immersion in distilled water for different periods. Normality of data distribution was tested by using the Kolmogorov–Smirnov test. Data were statistically in a model of repeated measures, using multivariate tests and Tukey’s multiple comparison tests at a significance level of p < 0.05.

**Results:**

∆E_00_ values of resin cements were influenced by cycle periods, significantly (p < 0.05). The highest ΔE00 values for long term were obtained in the NX3 (DC) (3.49 ± 0.87) and the lowest in the NX3 (LC) (1.41 ± 0.81). NX3 (LC), Variolink (DC), RELY X (LC) resin cements showed clinically acceptable color change after long-term aging (∆E_00_ < 1.8).

**Conclusion:**

Light-cure resin cements should be preferred for long-term color stability of full ceramic restorations.

## Background

Restorative materials like natural tooth color are largely used in dentistry to supply the esthetic requests of patients. Full ceramic restorations such as crowns, inlays, onlays and laminate veneers are mostly used treatment options in clinic and adhesive resin cements must be used for adhering these restorations to the natural teeth surface [1, 2]. According to polymerization type adhesive resin cements are classified as self-cure resins, dual-cure resins and light-cure resins. However adhesive resin cements were categorized total etch, self-etch and self-adhesive etch according to their content and bonding procedure during cementation [3–5].

Different cementing and bonding procedures and resin cements are required for each type of restoration [3]. Laminate veneers are an indispensable form of treatment for patients with esthetic expectancy. However, laminate veneers reflect the color of the resin cement as they are partially thin restorations. Laminate veneers are usually bonded to the natural tooth surface with light cure resin cements [6, 7]. But dual cure resin cements must be used when the laminate veneers are thicker than 2 mm and where the light cannot reach all over the restorations [7]. Composite resins are susceptible to color changes, which tend to become more visible when these materials are subjected to accelerated aging protocols. These changes are related to the oxidation of unreacted carbon double bonds or polymeric matrix, which leads to water diffusion and degradation of by-products [8]. The color change of resin cements after cementation in translucent restorations is a widespread esthetic problem and the esthetic restorations must be needed to repeat. Discoloration can develop depending on external and internal reasons. External factors include stains from food, drinks and cigarettes. Internal factors are directly related to resin chemical composition, filler type, resin matrix composite, photo initiator type, and polymerization type. The discoloration due to internal reasons is further increased by temperature changes in the oral environment [9–11].

Dual-polymerized resin cements contain tertiary amines and benzoyl peroxide, and over time degradation of color occurs. However, the color change of the photo-initiator camphorquinone, which is widely used in only light-polymerized resin cements, is much more insignificant. However, inadequate polymerization of camphorinon causes the discoloration to yellow shades. Some resin cements were produced photo-initiator tertiary amine free to prevent discoloration [12, 13]. It has been reported that new resin cements without tertiary amine show much less discoloration. In the clinic, discoloration was observed in the long-term and even in short term, depending on the chemical structure of the polymerized resin cements and internal factors [9]. In many studies, the color constancy of resin cements due to external factors has been studied, but in spite of the known influence of resin color on final restoration, only a few studies have centered upon resin cements themselves [14].

In order for restorations to be esthetically successful, an accurate and reliable method should be used in the evaluation of tooth color. In literature, in many studies evaluating color differences, it has been reported that the CIEDE 2000 system is more successful in detecting low color differences, although the most widely used system is CIE L*a*b and is considered sufficient [7–12]. CIEDE 2000 is based on CIE L*a*b and includes five fixes; these include the weighting functions of lightness, saturation (chroma) and hue, an interactive term (ΔR) between saturation and hue difference for blue colors. CIEDE 2000, developed to overcome the shortcomings of the CIE L*a*b system has been adopted by CIE as the new color system [7].

The aim of this research was to compare the long-term color stability of light-cure and dual-cure resin cements using an amine-free initiator system after aging. The null hypothesis of this study was that resin cements would effect the color stability of the type of polymerization and its chemical structure after long-term aging.

## Methods

This study design consisted of 60 specimens in each group at 95% power for statistical differences between groups according to power analysis with G power program 3.1.9.7 version. Sixty disk-shaped specimens were prepared from six different amine-free resin cements; NX3 Nexus light-cure (LC) and dual-cure (DC), Variolink Veneer (LC) and Variolink II (DC), Rely X Veneer (LC) and Rely X Ultimate (DC). The chemical contents and polymerization types of the specimens in groups are given in Table 1.

**Table 1:** Resin cements used in the study

Product	Group	Shade	Polymerization type	Initiator system	Content	Manufacturer
NX3	NXLC	Clear	Light cure	Free tertiary amines and benzoyl peroxide	Uncured methacrylate ester monomers, inert mineral fillers, activators and stabilizers, and radiopaque agent glycerine, water, fumed silica and inert glass powder, gelatin	Kerr, Canada
NX3 Dual Cure	NXLC	Clear	Dual cure	Free tertiary amines and benzoyl peroxide	HEMA, PTU, CHPO, uncured methacrylate ester monomers, titanium dioxide, and pigments	Kerr, Canada
Rely X Veneer	RXV	Translucent	Light cure	Distinct amines react with camphorquinone	BIS GMA and TEGDMA polymer	3 M Espe, USA
Rely X Ultimate	RXU	Translucent	Dual cure	Distinct amines react with benzoyl peroxide (redox polymerization system)	Methacrylate monomers, radiopaque silanated fillers, initiator, stabilizer, rheological additives. Catalyst paste: methacrylate monomers, radiopaque alkaline (basic) fillers, initiator, stabilizer, pigments, rheological additives	3 M Espe, USA
Variolink Veneer	VRV	Value 0	Light cure	Distinct amines react with camphorquinone	Di-methacrylates, inorganic fillers, ytterbium trifluoride, catalysts and stabilizers, pigments	Ivoclar-Vivadent, Liechtenstein
Variolink II	VRDC	Transparent	Dual cure	Distinct amines react with benzoyl peroxide (redox polymerization system)	Barium glass, ytterbium trifluoride, Ba-Al-F silicate glass, spheroid mixed oxide (%46 v, %70 w) BIS GMA, UDMA, TEGDMA	Ivoclar-Vivadent, Liechtenstein

Acrylic mold was used to create specimens from resin cements. Each specimen was constructed to be 1 × 10 × 10 mm dimensions, which corresponds to the spectrophotometer tip chamber, in accordance with ISO 7491:2000 standard. A total of 10 specimens were created for each group. The specimens were polymerized with a LED curing unit (GC-D-Light Pro LED, GC Corporation, Tokyo, Japan) for 40 s at four equal distances on specimen. The light irradiation for calibration was tested by a radiometer (Demetron L.E.D. radiometer, Sybron, Newport Beach, CA, USA) and verified for all groups at 850 mW/cm^2^. The specimens were finished using different grain of silicon carbide paper (#200, 400 and 600) to obtain 1 mm thick. The dimensions of the specimens were verified by a digital micrometer (Moore & Wright, Sheffield, UK). A CAD/CAM feldspathic ceramic block test specimen (VITA MARK II, VITA Zahnfabrik, Bad Säckingen, Germany) in 2M2C color was obtained. All specimens were kept in distilled water at 37 ± 2 °C for 24 h.

Specimens were first cleaned in ultrasonic cleaner for 10 min in distilled water and dried with oil-free air for 30 s. It was performed three times in the middle of the specimens with a specially calibrated spectrophotometer (VITA Easyshade, Vident, Germany) for color measurements. The data were obtained according to the CIE L*a*b color system. To simulate clinical situations, the CAD/CAM porcelain was placed without luting on all resin cement specimens for color measurement. And then, all specimens were aged with 30 s dwelling time between 5 and 55 °C for 5000 and 20,000 cycles.

After the aging process, color measurement of all specimens was made three times in the exact middle of the specimens with the help of a specially calibrated spectrophotometer (VITA Easyshade, Vident, Germany). The data were obtained according to the CIE L*a*b color system. To find the differences of the specimens with A2 color on the color scale, the calculation was made using the formula ΔE_00_.

$$\Delta E_{00} = \sqrt {\left( {\frac{{\Delta L^{\prime } }}{{k_{L} S_{L} }}} \right)^{2} + \left( {\frac{{\Delta C^{\prime } }}{{k_{C} S_{C} }}} \right)^{2} + \left( {\frac{{\Delta H^{\prime } }}{{k_{H} S_{H} }}} \right)^{2} + R_{T} \left( {\frac{{\Delta C^{\prime } }}{{k_{C} S_{C} }}} \right)\left( {\frac{{\Delta H^{\prime } }}{{k_{H} S_{H} }}} \right)}$$

Statistical analyzes were performed using the SPSS 22.0 program (SPSS Inc., Chicago, USA) program. Homogeneity of variance was examined by Kolmogrov–Smirnov test. The statistical analysis of the data obtained was performed by one-way ANOVA with repeated measure design method in the data providing parametric distribution (p < 0.05). Tukey’s HSD test was applied for multiple comparisons (p < 0.05).

## Results

The one-way ANOVA with repeated measure results of the samples is shown in Table 2.

**Table 2:** The one-way ANOVA with repeated measure results

Source	Tests of between-subjects effects					
	Type III sum of squares	df	Mean square	F	Sig	Partial eta squared
Intercept	518.597	1	518.597	2890.177	0.000	.972
Groups	44.952	5	8.990	50.104	0.000	.749

A significant difference was found between cement groups in terms of ∆E_00_. The mean ∆L, a, b values before and after the accelerated aging for all the groups are shown in Table 3.

**Table 3:** Mean ∆L, ∆a, ∆b values before and after aging

Thermal aging				
Test specimens		Start	5000 cycles	20,000 cycles
NX3 (LC)	L	84.64	84.27	81.81
	a	− 0.27	− 0.09	− 0.37
	b	6.76	6.62	6.53
NX3 (DC)	L	85.90	85.15	81.40
	a	− 1.06	− 0.33	− 1.04
	b	6.84	6.43	7.17
Variolink (LC)	L	83.87	85.43	83.02
	a	2.19	2.17	2.35
	b	7.97	8.27	7.86
Variolink (DC)	L	85.57	84.18	86.26
	a	− 0.16	− 0.01	− 0.47
	b	5.77	5.57	6.57
RelyX (LC)	L	86.70	85.95	87.76
	a	− 0.60	− 0.65	− 0.06
	b	4.72	5.24	5.42
RelyX (DC)	L	84.71	85.29	82.41
	a	− 0.33	− 0.37	− 0.13
	b	7.47	7.36	6.78

The ∆E_00_ values for the color after polymerization and 5000 thermal cycles (∆E_00_1), between 5000 and 20,000 thermal cycles (∆E_00_2), between polymerization and 20,000 thermal cycles (∆E_00_3) are given Table 4.

**Table 4:** ∆E_00_ values before and after thermal cycles

	ΔE_00_1	ΔE_00_2	ΔE_00_3
NX3 light cure	0.37 ± 0.01	2.14 ± 0.72*	1.41 ± 0.81
NX3 dual cure	0.56 ± 0.01	2.80 ± 0.63*	3.49 ± 0.87*
VAR light cure	0.63 ± 0.05	1.06 ± 0.32	1.99 ± 0.56
VAR dual cure	0.94 ± 0.08	0.87 ± 0.08	1.53 ± 0.46
RELYX light cure	0.65 ± 0.05	1.24 ± 0.20	1.47 ± 0.40
RELYX dual cure	0.40 ± 0.01	1.48 ± 0.36	1.91 ± 0.50

*Statistical difference between values

**Fig. 1:**
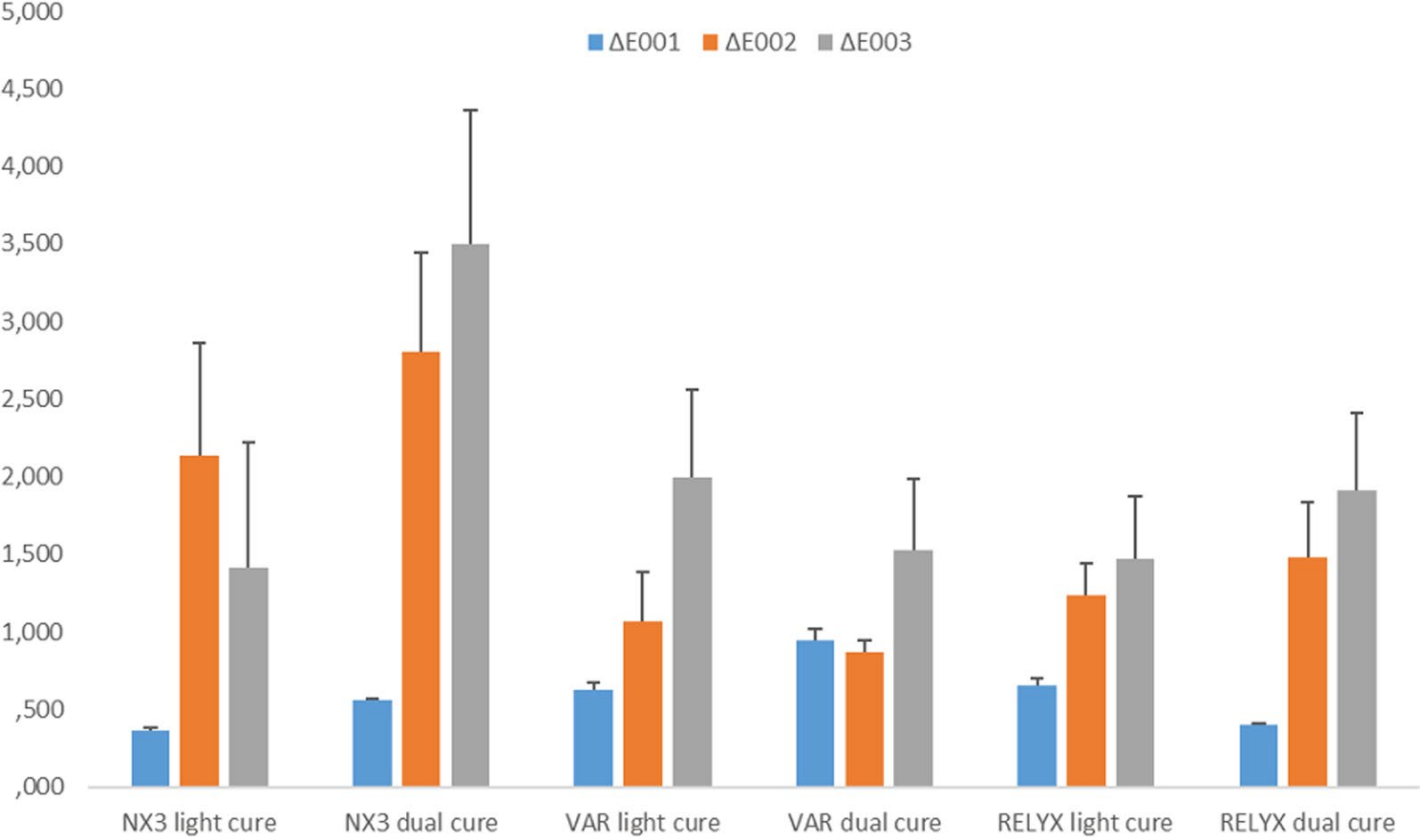
Bar chart showing ∆E_00_ values before and after thermal cycles

There was a significant difference between ∆E_00_1 and ∆E_00_2 − ∆E_00_3 values statistically when the effects of aging on the color changes were evaluated (p < 0.05). The highest ∆E_00_1 values were obtained from the VAR (DC) group (0.94 ± 0.08) while the lowest values in the NX3 (LC) group (0.37 ± 0.01). The highest ∆E_00_2 values were obtained from the NX3 (DC) group (2.80 ± 0.63) while the lowest values in the VAR (DC) group (0.87 ± 0.08). The highest ΔE_00_3 values were obtained in the NX3 (DC) (3.49 ± 0.87) group and the lowest in the NX3 (LC) (1.41 ± 0.81) group. ∆E_00_ differences of the examples are given in the bar graph table in Fig. 1.

## Discussion

In this study, long-term color stability of different amine-free light-cure and dual-cure resin cements was evaluated after aging and long-term color changes of resin cements were observed significantly. The hypothesis was confirmed.

The long-term color stability of resin cement is important for the esthetics of laminate veneers, all-ceramic crowns. The color change of the cement under restorations such as full ceramic and porcelain laminate veneer can be reflected from the restoration, which effects the esthetic appearance of the restoration. Therefore, the cement's color stability is one of the most important clinical factors in the success of such restorations [15–18].

Adhesive resin cements are used not only for gluing esthetic restorations, but also for the final color of the restoration [17, 18]. Researchers reported that the adhesive resin cement used under ceramic restoration was effective on the final color of the restoration. It should not be considered that only the full ceramic restoration will determine the final color of the restoration to be cemented; It is known that the type and thickness of porcelain used as restorative material on this final color, the color and thickness of the resin cement, as well as the color of the dental tissue under it [19, 20]. Miyasaka et al. [21] reported that one of the biggest disadvantages of polymeric materials in their studies is that they show color change over time and even restorations need to be renewed due to this change. Terry reported that color discordance is one of the most important reasons for changing the restorations applied to the anterior region [22].

In resin cements, a low degree of polymer transformation can cause discoloration. Acidic monomers remaining on the tooth surface after etching can react with amines to inhibit the redox reaction of the resin. This reaction depends on the amount of acid and the degree of acidity [23]. Additionally, chemical differences in resin components such as the purity of oligomers and monomers, the concentration and type of activators, initiators, inhibitors, oxidation of unreacted double bonds between carbon and carbon, and fillers can effect the color stability [24]. Differences in color variation between brands may be due to filler particle differences and degradation of polymer material [25]. It is very difficult for the human eye to distinguish small color differences in dental materials. In studies conducted to numerically express the visible color change, there is no exact precision in determining the limit that can be noticed by the human eye [26, 27]. According to some researchers ΔE value that cannot be noticed by the human eye for all-ceramic restorations was 1.6, while Ragain et al. reported the E value as the limit where color difference was not acceptable clinically in their study. ADA (American Dental Association) declared the tolerance limit of ΔE units of color scales as 2 [28]. In the last decade, the detectability and acceptability threshold values for the CIELAB system have been reported as 1.2 and 2.7, respectively, and the detectability and acceptability threshold values for the CIEDE2000 system as 0.8 and 1.8, respectively [29]. For CIEDE2000, the acceptable value of ∆E_00_ is 1.8, while the noticeability value of ∆E00 is 0.8. The CIEDE2000 formula is better suited for color difference calculations, as it detects even the slightest differences in tooth colors and provides better indications [30]. Janda et al. [31] showed that the prevalence of coloration depends not only on polymerization time, aging conditions and material, but also on polymerization mode.

The color stability of composites depends on various factors, such as the polymerization mode, polymerization time and the composition of the material. The researchers' results showed that color stability was influenced by polymerization time, aging conditions, and the composition of the material being tested. Despite the same polymerization mode, the same polymerization time application and the use of the same light device, it is thought that the resin cements show a different degree of color change in the study, as researchers pointed out, may be due to the composition of the materials [2, 32].

Turgut and Bagis reported that the color or color coordinate change that occurred after polymerization is related to the color of the material. It was found that the brand and colors of composite resins were significantly effective in color changes during light polymerization. The effects of polymerization time and color tone on color changes of light-curing resin compounds were investigated. Lighter or less chromatic colors showed more color changes than more chromatic or darker colors [33].

In the studies on color changes of resin composites after polymerization, it has been reported that remarkable color changes occur and the size of the color change varies according to the properties of the material. A statistically significant relationship was found between the colors and brands of composite resins in terms of color change during polymerization with light [2].

When the studies are examined, it is seen that various aging techniques are used in the evaluation of the color changes of dental materials. These; thermal cycle, water storage, photoaging and accelerated aging [34]. In our study, color change was observed in all resin cement groups after thermal aging. Dual-cure resin cements showed the most color change over time. Magalhães et al. reported that Bis-GMA monomers caused the yellowing of resin cements after exposure to ultraviolet light and heat in the study where cemented porcelain laminate and color change were examined. It has also been stated that, as composite-based materials age, resin monomers can cause changes in color stability due to their water absorption properties [35].

In some studies, conducted with Variolink II, Variolink Veneer, which were previously produced by the same company and used frequently clinically, with a similar content to Variolink (LC and DC) used in this study, no significant difference was found between LC cements and DC cements in terms of color change [33, 36]. The reason for this difference is that the ratio of light-sensitive molecules in Variolink II resin cement is higher compared to other DC resin cements [36]. DC cements become more reliable in terms of color stability as the proportion of light-sensitive molecules they contain increases. It is also claimed by the manufacturer that the photosensitivity and photo-reactivity of the reaction initiator in the Variolink resin cement used in this study are superior to previously developed systems [12, 37].

In the study where 0.5 mm thick porcelain laminates are cemented with 3 different cements (Rely X Unicem, Rely X Ultimate and Rely X Veneer), the specimens were subjected to accelerated thermal aging and color changes were examined. In the study in which color measurements were made by spectrophotometer 24 h after cementation and after 1000, 2000, 3000 thermal cycles aging, the color difference (∆E) in all specimens increased but there were no statistically significant differences and all of them were in the clinically acceptable range after 3000 thermal cycles (∆E < 3.5) [38]. In this study, a clinically acceptable color change was observed in Rely X (LC and DC) short-term aging (∆E_00_ < 1.8). Rely X Ultimate (DC) exhibited color change above the clinically acceptable limit while Rely X Veneer (LC) showed a clinically acceptable color change in long term aging (∆E_00_ < 1.8).

In the study of Kılıc et al. where the porcelain veneers, which were cemented with light cure and dual cure cements, were subjected to rapid aging in the thermal cycle device, examined the color difference that occurred after the light cure cements showed less color changes, found statistically significant differences, but the color change in all cements was clinically accepted. They reported that they were within the limits, therefore, each type of cements was clinically successful [39]. Pissaia et al. investigated the color change of NX3 (LC and DC) resin cement in 6 months and 3 years in their study. They observed that NX3 (LC) cement was above the clinically acceptable limit at 6-months color change, but NX3 (DC) cement was at the clinically acceptable limit. In the long term, they observed that both cements showed high color change (∆E = 3.5) [40]. Parallel results were obtained in this study. It can be attributed to the fact that NX3 (DC) cement contains HEMA, PTU, CHPO that can absorb water in higher ∆E_00_.

Both intrinsic and extrinsic factors are effective in resin cement coloring. The chemical content of the material as the intrinsic factor, the degree of water absorption, the polymerization type, etc. It is effective. Food, drinks, cigarettes etc. as extrinsic factors. It is effective. Especially esthetically, the coloring of the resin material in the anterior region results in esthetically negative results [2, 24].

Kim et al. reported that color stability is directly related to the resin phase of composite resins [41]. Falkensammer et al. reported that, under changing physicochemical conditions, the color stability of resin composites can be improved by using less water absorption of materials, higher filler-to-resin ratio, reduced particle sizes, and the use of the optimal filler-matrix system [42].

## Conclusions

Considering the limitations of this study, the following conclusions were drawn:

1. Light-cure resin cements showed lowest color changes in long-term after thermal aging;
2. However, dual cure resin cements could not show clinically acceptable color stability in the long-term because of chemical content and behavior;
3. In the long-term, light-curing resin cements firstly could be preferred in order for the color change to be considered clinically successful in esthetic regions.

## Data Availability

The datasets used and/or analysed during the current study are available from corresponding author on reasonable request due to privacy reasons and large data size.

## Abbreviations

DC: Dual-cure
LC: Light-cure
HEMA: Hydroxyethyl methacrylate
PTU: Pyridyl thiourea
CHPO: Cumene hydroperoxide
BIS-GMA: Bisphenol-A-diglycidyl ether dimethacrylate
TEGDMA: Triethylene glycol dimethacrylate
